# Design, Implementation and Environmental Impact of Cutoff Wall for Pollution Control in an Industrial Legacy Site

**DOI:** 10.3390/toxics13010011

**Published:** 2024-12-25

**Authors:** Lu Yu, Sichen Chen, Jinnan Wang, Zhihong Zhang, Yan Huang

**Affiliations:** 1 College of Materials Science & Engineering, Beijing University of Technology, Beijing 100124, China; chensien0@163.com (S.C.); wjn2158@163.com (J.W.); 2 Faculty of Architecture, Civil and Transportation Engineering, Beijing University of Technology, Beijing 100124, China; 3 Zhejiang Zone-King Environmental Sci & Tech Co., Ltd., Hangzhou 310064, China; yanhuang_now@163.com

**Keywords:** organic pollutants, heavy metals, cutoff walls, soil–groundwater environment, pollution control and prevention, numerical simulation

## Abstract

Heavy metal-organic pollutants compound pollution at industrial legacy sites and have caused damage to the ecological environment and human health during recent decades. In view of the difficulty and high cost of post-contamination remediation, it is worth studying, and practically applying, cutoff walls to reduce the spread of pollution in advance. In this study, field-scale studies were carried out at e-waste dismantling legacy sites in Taizhou, Zhejiang Province of China, through the process of site investigation, numerical simulation, and cutoff wall practical application. Firstly, the concentrations and spatial distributions of Pb, Cd and polychlorinated biphenyls (PCBs) and poly brominated diphenyl ethers (PBDEs) were identified in both soil and groundwater. Then, potential dispersal routes of key combined contaminants (Pb and PCBs) at the soil–groundwater interface were systematically studied through numerical simulation applying Visual MODFLOW-MT3DMS. One site was chosen to predict the barrier effect of differently sized cutoff walls based on the migration path of compound pollutants. A protocol for a cutoff wall (50 m length × 2 m width × 3 m height) was finally verified and applied at the real contaminated site for the blocking of compound pollutant diffusion. Further, the groundwater quality of the contaminated site was monitored consecutively for six months to ensure the durability and stability of barrier measures. All pollutant indicators, including for Pb and PCB complex pollutants, were reduced to below the national Grade IV groundwater standard value, achieving environmental standards at these polluted sites and providing possibilities for land reuse. In summary, this field-scale test provided new ideas for designing cutoff walls to block the diffusion of complex pollutants; it also laid a basis for the practical application of cutoff walls in pollution prevention and control of complex contaminated sites and for soil–groundwater environmental protection at industrial heritage sites.

## 1. Introduction

In recent decades, with the promulgation and implementation of environmental protection policies, most heavy industries have been relocated or banned. However, the compound pollution environmental problem of urban industrial legacy sites still exists, which can cause damage to the ecological environment and human health, as well as to economic and social development. The environmental pollution problem at industrial sites, especially the combined pollution problem, has become one of the main constraints for urban environmental management and land resource intensification.

E-waste was once regarded as a valuable and recyclable resource in China, inducing a large e-waste dismantling industry, which gradually formed in coastal areas, including eastern China and the Pearl River Delta Region. These areas possessed sites for e-waste dismantling and disposal sites during recent decades [1] (Lin et al., 2022a), which significantly increased local economic development by offering more job opportunities [2] (Widmer et al., 2005). However, these e-waste disposal activities damaged the local ecological environment and posed a threat to human health.

The Chinese government began to ban the import of e-waste in 2018 to eliminate the environmental problems caused by its dismantling and disposal. Even so, the problem of contamination of the sites which had once engaged in the electronic dismantling industry still existed. In particular, soil inorganic–organic compound pollution was reported in the surrounding areas of informal dismantling factories or family handicraft workshops [3] (Bakhiyi et al., 2018). For instance, compound pollution of heavy metals (Pb, Cd, Cr, Cu, Ni, etc.) and organic pollutants (PCBs, PBDEs, PAHs, etc.) has existed for a long time [4,5,6] (Shen et al., 2008, 2009; Tang et al., 2010). Moreover, local food products (fish, rice, vegetables, seafood, chicken, and eggs) accumulated high levels of toxic substances (PCBs, PBDEs, Cd, Pb, Cu, As, etc.), which could expose residents to potential health risks [7,8,9,10,11] (Xing et al., 2010; Zheng et al., 2023; Awasthi et al., 2018; Wang et al., 2021; Lin et al., 2022b). The incalculable environmental and health problems caused by crude e-waste dismantling may even last for generations. Pollution at the soil–groundwater interface was even more severe and complex, affected by industrial waste accumulation [12] (Jia et al., 2020), rainfall [13,14] (Gu et al., 2010; Liu et al., 2024), overland runoff [15] (Xia et al., 2022) and multiple media diversity [16,17] (Xue et al., 2023; Xiang et al., 2024). Therefore, it is urgent to pay attention to the environmental problems of legacy sites and to formulate effective and convenient solutions.

During recent years, some attention has been paid to compound pollution, which was widespread in typical e-waste disposal sites, e.g., Guiyu town in Guangdong Province [18,19] (Leung et al., 2007; Li et al., 2018), Taizhou city in Zhejiang Province [4] (Shen et al., 2008), etc. However, most studies focused on contamination distribution [12,20] (Xu et al., 2019; Jia et al., 2020), the hazards of pollutants in soil–groundwater systems [1,21] (Lin et al., 2022a; Zhai et al., 2022), and environmental remediation techniques [1,22,23] (Yang et al., 2020; Lin et al., 2022a; Wu et al., 2022), rather than planning a targeted program for implementation in order to prevent pollution generation and block pollution diffusion.

The United States [24,25] (US EPA, 2012, 2013), the European Union [26] (UK EA, 2009) and other countries or regions have long taken pollution control as a top priority of site management [25,27] (US EPA, 2013; UK EA, 2016). Comparatively, prior pollution control technology (especially engineering control) to stop the spread of pollution was of little concern in China before this current decade, when compared to post-environmental remediation technology. In July 2014, the Ministry of Environmental Protection of the People’s Republic of China introduced technical guidelines for contaminated sites, which provided a theoretical and implementation basis for beginning the work of site environmental governance and pollution control. Even so, compared with restoration work post-pollution, systematic work involving site pollution prevention and control and management is far from enough. Pollution control is broadly considered to consist of two categories: system management and engineering control. System management involves administrative, legal and economic means, while engineering control focuses on the use of specific technical means to reduce the discharge of pollutants. The most important and critical step is that of engineering control technology at the point at which the cross-media pollution control of the complex contaminated site is carried out.

Therefore, we have studied the blocking mechanism and the possibility of practical application of cutoff walls (within the scope of engineering control) to prevent the diffusion of complex pollutants. Firstly, typical e-waste dismantling legacy sites in Taizhou, Zhejiang Province, China, were selected. The main pollutants in soil and groundwater at these sites and their migration rules were systematically studied and grasped by applying the Geographic Information System. Secondly, one site was chosen to study the pollutant migration path and to predict the barrier effect of cutoff walls by applying numerical simulation. Thirdly, the real effect and the persistence of the cutoff wall blocking pollutants were verified through site application. Finally, groundwater monitoring was carried out for over six months. The combination of numerical simulation and field tests contributed to a more perfect cutoff wall scheme and implementation to block the diffusion of heavy metal–organic pollutant compound pollution; while six-month groundwater monitoring ensured the durability and stability of the cutoff wall measures. This research was expected to clarify the pollution resistance and control mechanism of the cutoff wall acting on the combined pollution environment at the industrial legacy site and to provide a theoretical basis and a practical reference for environmental treatment. 

## 2. Materials and Method

### 2.1. Study Area Description

**Figure 1:**
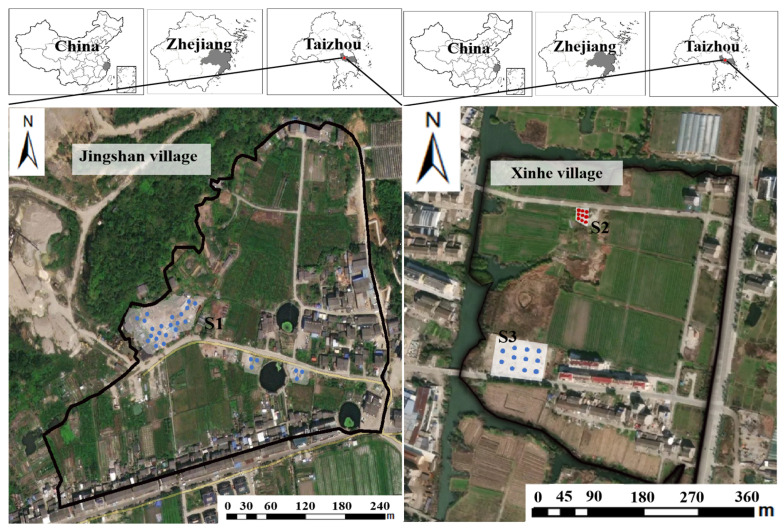
Three representative e-waste dismantling legacy sites in Taizhou city: site 1 is in Jinshan village, while site 2 and site 3 are in Xinhe village. site 1, translucent grey shading blue dots represent sampling points; site 2, red dots on the white background represent sampling points; site 3, blue dots on the white background represent sampling points.

The city of Taizhou in Zhejiang Province, China, has been engaging in e-waste dismantling for several decades. Three representative dismantling legacy sites in this city (Figure 1) were selected for soil–groundwater pollution investigation and physicochemical analysis. One was Jingshan village in the Luqiao District (site 1), located at the foot of a deserted mountain (E121.36, N28.54), with a long history of e-waste disassembly [1] (Lin et al., 2022a). The other two sites (site 2 and site 3) were at Xinhe village in Zeguo Town (E121.36, N28.49), with one (site 2) located in the north of the village, where silt and sludge excavated from the river had accumulated; another (site 3) was located in the southwest of the village, which has a manual disassembly workshop in front of and behind the villagers’ houses. The annual precipitation for these three sites in Taizhou City was 1185–2029 mm; the temperature was 16–23 °C.

### 2.2. Field Research and Sampling

Each site was divided into small grids of 10 m × 10 m. A total of 27 sample points for site 1, 11 sample points for site 2, and 9 sample points for site 3 were used for collection in September 2021. The topsoil samples were collected at a depth of 10–30 cm, while the deep soil samples were collected at a depth of 1.00–3.60 m. The groundwater samples were collected at a depth of 1.04–4.80 m.

The basic information on hydrogeological condition in each site was fully gathered through field investigation (Table 1). Soil particle composition was 13.1% silt, 80.4% sand and 6.5% clay in site 1; 42.1% silt, 7.6% sand and 50.3% clay in site 2; and 8.4% silt, 86.6% sand and 5.0% clay in site 3. Soil textures were Ultisol in site 1, Miscellaneous Fill in site 2, and Anthrosol in site 3. Soil organic matter was 56.3 g/kg in the topsoil and 2.83 g/kg in the deep soil of site 1; 40.8 g/kg in the topsoil and 2.97 g/kg in the deep soil of site 2; and 61.2 g/kg in the topsoil and 49.0 g/kg in the deep soil of site 3. Dissolved organic matter was 51.5 mg/kg, 41.89 mg/kg and 53.49 mg/kg in site 1, site 2 and site 3, respectively. Vertical hydraulic conductivity (K_v_) was 4.20 × 10^−8^ cm/s, 1.19 × 10^−7^ cm/s and 3.21 × 10^−8^ cm/s in site 1, site 2 and site 3, respectively; while horizontal hydraulic conductivity (K_h_) was 4.48 × 10^−8^ cm/s, 9.61 × 10^−8^ cm/s and 3.85 × 10^−8^ in site 1, site 2 and site 3.

### 2.3. Chemical Analysis

#### 2.3.1. Heavy Metal Concentration Test

Soil samples from three sites were air-dried, ground, sieved (2 mm mesh), and acid digested according to national soil pollution conditions detailed analysis of soil sample test technical provisions method [28] (Ministry of Ecology and Environment of the People’s Republic of China, 2017) and then determined in the laboratory by inductively coupled plasma mass spectrometry (ICP-AES, Optima8000, PerkinElmer, Waltham, MA, USA) for the determination of heavy metals including Cd, Cr, Cu, Ni, Pb and Zn. Quality control and assurance were conducted by preparing the same procedures for blank value determination, parallel determination experiments and mixed certified reference materials (Pb, GBW(E)083779; Cd, BWB2078-2016; Cr, BWB2142-2016; Cu, BWG083396-50-1; Zn, GBW(E)083392; Ni, GBW(E)083786). It was also ensured that the analytical process was within the quality control limits. The comparison with the relative standard deviation (RSD) showed that the analytical deviation was less than 3%. To determine whether the above pollutant indicators of the topsoil exceed the standard, follow the pollution risk control standard of Site environmental quality-Risk control standard for soil contamination of development land GB36600-2018 [29], with standards in Cd, Cr, Cu, Ni, Pb, Zn were 47, 30, 8000, 600, 800, 3500 mg kg^−1^, respectively.

#### 2.3.2. Organic Pollutant Concentration Test

Soil PCBs and PBDEs were extracted, purified and determined according to test criteria [30,31] (HJ 743-2015; US EPA, 2018) and published references [32,33] (Li et al., 2021; Xiang et al., 2016). Detailed procedures were provided in Supporting Information. In total, 42 PCB congeners (Aroclor 1242) and 15 BDE congeners (BDE-28, 47) were quantified in the soil samples using an external standard solution. The recoveries of PCBs and PBDEs in soil were 90–112% and 85–121%. The standard value of PCBs was 1400 μg kg^−1^, referred from the pollution risk control standard of Site environmental quality-Risk control standard for soil contamination of development land GB36600-2018 [29]; And the standard value of PBDEs referred to Regional Screening Levels (RSLs)-US EPA with 780 μg kg^−1^ (Carcinogenic SL).

### 2.4. Numerical Simulation

In order to understand how heavy metal-organic pollutants existed and diffused at the soil–groundwater interface of the above e-waste dismantling legacy sites, we applied numerical simulation for delineating the dispersal path of contaminants as well as predicting the effectiveness of cutoff walls. After basic chemical analysis, site 2 in Xinhe village was chosen for further study.

#### 2.4.1. Groundwater Flow Model and Solute Transport Model

Visual MODFLOW 4.6 (VM) is the most commonly used simulation software for describing groundwater movement and pollutant transport. Based on the grid finite difference method, the water equilibrium equations of each grid in the study area were established. The solute transport model MT3DMS in VM software was used to simulate the water conducting space and storage space.

#### 2.4.2. Model Building and Site Environment Simulation

(1) Boundary condition setting

Since the concentrations of Pb and PCBs at the soil–groundwater interface of the above sites could exceed the standards, Pb and PCBs were used as characteristic pollutants for transport simulation. Site 2 had a history of centralized e-waste disposal, which was reported that Pb and organic pollutants were at risk of exceeding or exceeding the standard to varying degrees in soil and groundwater. Therefore, site 2 was chosen for further study to identify the transfer law of complex pollutants.

When VM was used to simulate solute transport, specific pollution indexes were included in the model to assign the initial concentration for simulation. In the VM software, the concentration of Pb was set as 10 mg L^−1^ and the concentration of PCBs was 0.50 mg L^−1^ according to the pollution survey data from site 2. Regardless of whether Pb or PCBs was the main pollutant in the combined pollution, the solute transport model was adopted for a ten-year simulation study with different time nodes as 1 day, 365 days, 730 days, 1825 days, and 3650 days.

Site 2 was surrounded by water on three sides and land on one side. Soil structure was silt to clay with low permeability coefficient (Figure S1), the groundwater of study area was mainly distributed in two layers: the upper layer belonged to pore phreatic layer with shallow, and the lower layer belonged to bedrock fracture water. The buried depth of the water table was about 1.04–4.80 m, and the annual variation of the water table was between 1.00–2.00 m. The terrain in and around the study area was flat without obvious watershed, and the groundwater level changes steadily over the years. Groundwater flow map indicated that groundwater flow from west to east or from southwest to northeast of contaminated site in different seasons (Figure S2). The groundwater discharge mode in the non-study area on the east side was the same, and there was no obvious groundwater flow interaction on the east side, so the water isolation boundary was located.

Pollutants transport law in the groundwater, as well as the effect of cutoff walls on pollution transport were both simulated in the VM software. The simulation time was set to 10 years (3650 days), and the initial concentration of Pb was set to 10 mg L^−1^ while the initial concentration of PCBs was set to 0.50 mg L^−1^ based on previous site investigation.

(2) Model building

In order to predict and evaluate the control effect of vertical cutoff wall on pollution sources, the scope of numerical simulation was consistent with the solute transport model of groundwater without any measures (Figure S3A). Therefore, the grid was divided by finite difference discrete method in this study. Site 2 was divided into 60 rows and 43 columns with the size of each grid 10 m × 10 m. The finite difference mesh in the contaminated area and the area around the cutoff wall was refined (20 times the degree of segmentation) so as to improve the accuracy of the simulation (Figure S3B).

The water exchange between groundwater and rivers, as well as the impact of vertical rainfall and evaporation on groundwater were considered in this saturated groundwater system with unstable flow. However, the impact of artificial water intake on groundwater was not considered since the amount of artificial water intake was relatively small. The specific stratigraphic division was shown in Figure S3, where the wavy band on the top represented the distribution of rivers around site 2, while the effective area grids were divided into 6 layers using different shades of color.

(3) Parameter calibration and optimization

According to the hydrometeorological data of the site, the initial parameters of the model were entered to set up the groundwater flow model. By comparing the changes of the measured water level and the simulated water level in the monitoring well (Figure S4), the groundwater flow model had a good degree of fitting level with standard mean square error was 4.977% and the correlation coefficient was 0.993. The results showed that the model has a good fitting accuracy and could truly reflect the dynamic change trend of the groundwater level in the study area.

### 2.5. Design and Implementation of Cutoff Walls

Cutoff walls were designed as follows: wall 1—50 m × 1 m × 3 m (length × width × height) or wall 2—50 m × 1.5 m × 3 m or wall 3—50 m × 2 m × 3 m or wall 4—55 m × 1.5 m × 3 m according to parameters of pollution plume area and groundwater barrier depth. At first, VM software was applied to predict the effects of different sizes of cutoff walls blocking pollutants migration in the groundwater media. Then, the most effective scheme was implemented in the polluted site to hinder the transport of Pb-PCBs compound pollutants. In addition, six wells around cutoff walls were set up to facilitate regular groundwater quality monitoring and chemical testing (Figure S5). During this six-month monitoring period, if the concentration of potential pollution plume did not exceed the environmental quality standard of construction land, it indicated that cutoff walls were appropriate and effective.

## 3. Results and Discussion

### 3.1. Characteristics of Heavy Metal-Organic Pollutants in Soil and Groundwater

**Figure 2:**
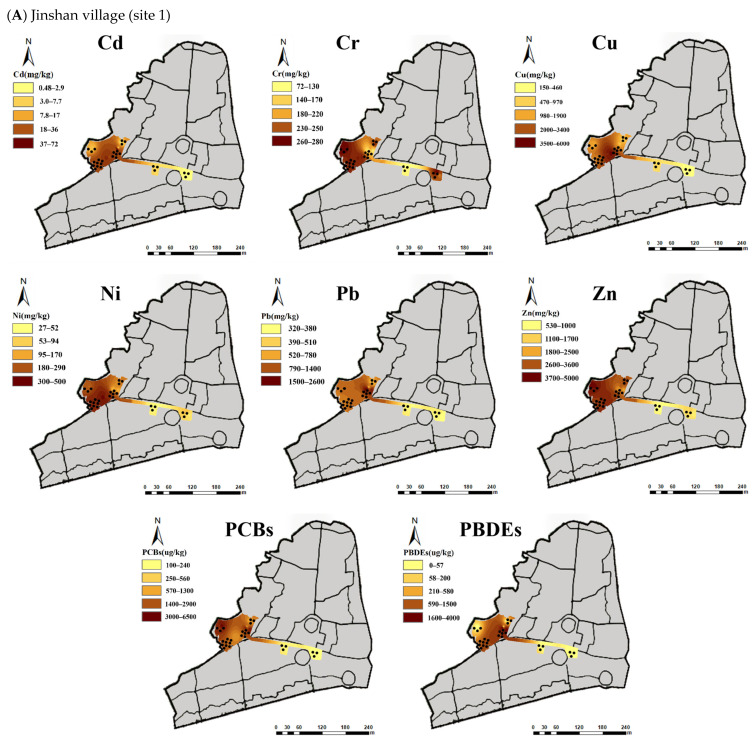

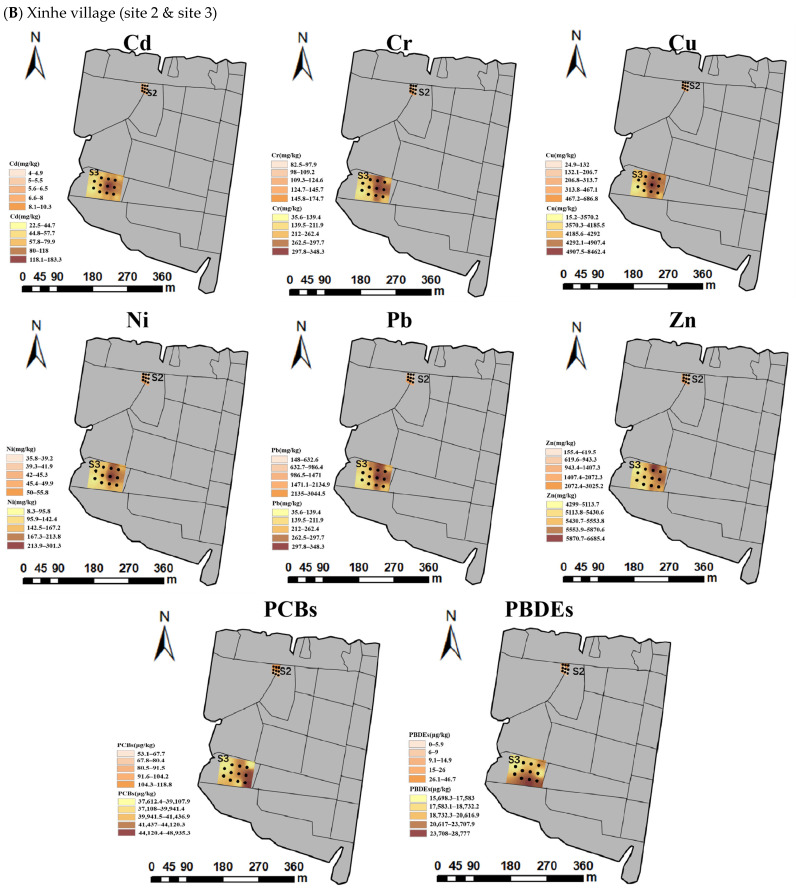
Concentrations of main pollutants in the topsoil of three representative e-waste dismantling legacy sites: the darker the color, the higher the concentration. (**A**). site 1 in Jinshan village; (**B**). site 2 and site 3 in Xinhe village. Pollutants included heavy metals (Cd, Cr, Cu, Ni, Pb and Zn) and organic pollutants (PCBs, PBDEs). To determine whether the above pollutant indicators of the topsoil exceed the standard, follow the pollution risk control standard of [29] Site environmental quality-Risk control standard for soil contamination of development land GB36600-2018, with standards in Cd, Cr, Cu, Ni, Pb, Zn were 47, 30, 8000, 600, 800, 3500, respectively, and the standard in PCBs was 1400 μg kg^−1^; And the standard value of PBDEs referred to Regional Screening Levels (RSLs)-US EPA with 780 μg kg^−1^ (Carcinogenic SL).

Pb concentration of the topsoil in *Jinshan* village exceeded the soil pollution risk control standard [29] (Site environmental quality-Risk control standard for soil contamination of development land GB36600-2018) of construction land by 0.06–0.75 times (Figure 2A), while which in Xinhe village exceeded by 5.24 times (Figure 2B). Cd concentration of the topsoil in Xinhe village exceeded the soil pollution risk control standard by 0.82 times (Figure 2A). Other heavy metal pollutants in the two villages did not exceed the standard value.

The concentration of PCBs in the topsoil exceeded the soil pollution risk control standard of construction land by 0.33–2.75 times in Jinshan village (Figure 2A) and 28.3 times in Xinhe village (Figure 2B). The concentration of PBDEs in the topsoil exceeded the standard of construction land by 1.47–4.07 times in Jinshan village (Figure 2A) and 25.5 times in Xinhe village (Figure 2B).

**Figure 3:**
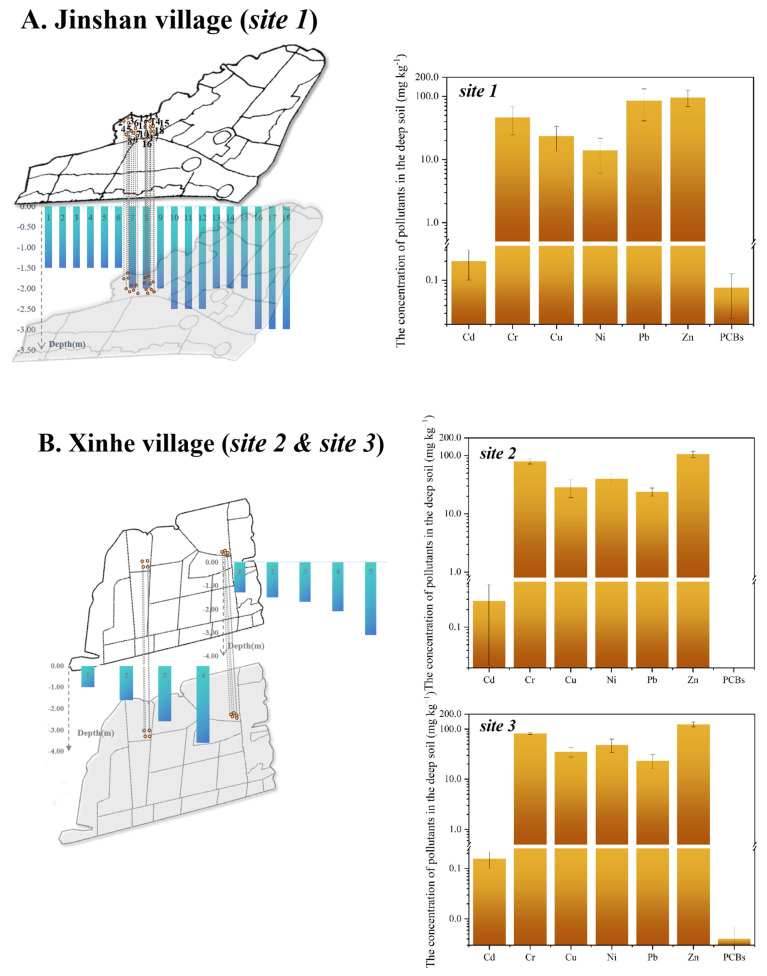
Concentrations of main pollutants in the deep soil of three representative e-waste dismantling legacy sites. (**A**). site 1 in Jinshan village; (**B**). site 2 and site 3 in Xinhe village. The blue-green bar chart represented the sampling depth of the deep soil, where site 1 was 1.5–3.0 m underground, site 2 was 1.3–3.1 m underground, and site 3 was 1.0–3.6 m underground. The yellow-brown bars represented the concentrations of pollutants. Pollutants included heavy metals (Cd, Cr, Cu, Ni, Pb and Zn) and organic pollutant (PCBs). The concentration of PBDEs in the deep soil of above three sites was very low and negligible.

Eighteen samples of deep soil in Jinshan village (site 1) were taken for heavy metals and organic pollutants test, while five samples of site 2 and four samples of site 3 in Xinhe village were taken for determination of deep soil contaminants. Results indicated that neither heavy metal nor PCBs exceeded the control value in both Jinshan village and Xinhe village (Figure 3), even though Pb concentration (86.0 mg kg^−1^) in the deep soil had the risk of exceeding the screening value.

**Figure 4:**
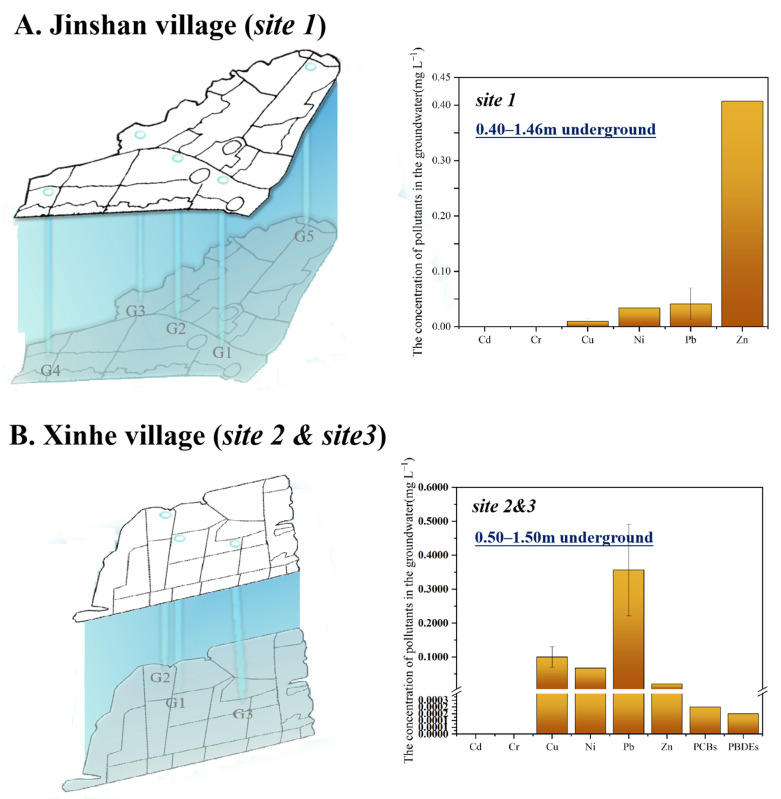
Concentrations of main pollutants in the groundwater of three representative e-waste dismantling legacy sites. (**A**). site 1 in Jinshan village; (**B**). site 2 and site 3 in Xinhe village. The light-blue hollow circle represented the sampling site of the groundwater while the light-blue vertebrae represented the specific sampling depth, where site 1 was 0.40–1.46 m underground, and site 2 and site 3 were 0.50–1.50 m underground. The yellow-brown bars represented the concentrations of heavy metals (Cd, Cr, Cu, Ni, Pb and Zn) and organic organic pollutants (PCBs and PBDEs). PCBs and PBDEs in site 1 groundwater were below detectable limit.

As for pollutant concentrations of groundwater in the above three sites, Pb concentration of samples in G2 (southwest of site 3) and G3 (south of site 2) in Xinhe village (Figure 4) exceeded water quality Grade IV standard value of China’s national groundwater quality standard [34] (GB/T 14848-2017) by 0.63 times and 2.56 times. Indexes of other heavy metals and organic pollutants were not exceeding the groundwater Grade IV standard in the above three sites. Nevertheless, Ni concentration was close to the limit of Grade IV standard value, and Ni exceeded the limit of Grade III standard value by 2.33 times. Moreover, the concentration of PCBs was 40% of the Grade III standard value. Considering the spatial heterogeneity of site sampling, pollutant indicators such as Ni and PCBs, just like Pb (which significantly exceeded the standard), should be given continuous attention.

### 3.2. Transport of Heavy Metal-Organic Pollutants at the Soil-Water Interface

**Figure 5:**
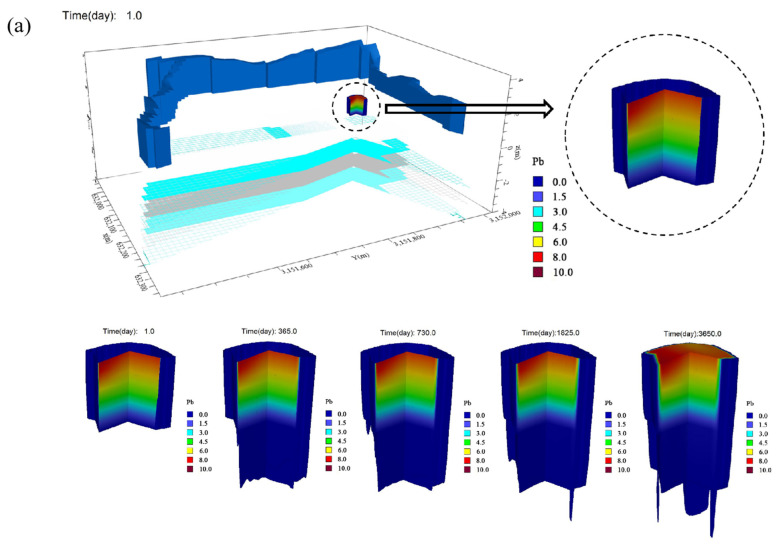

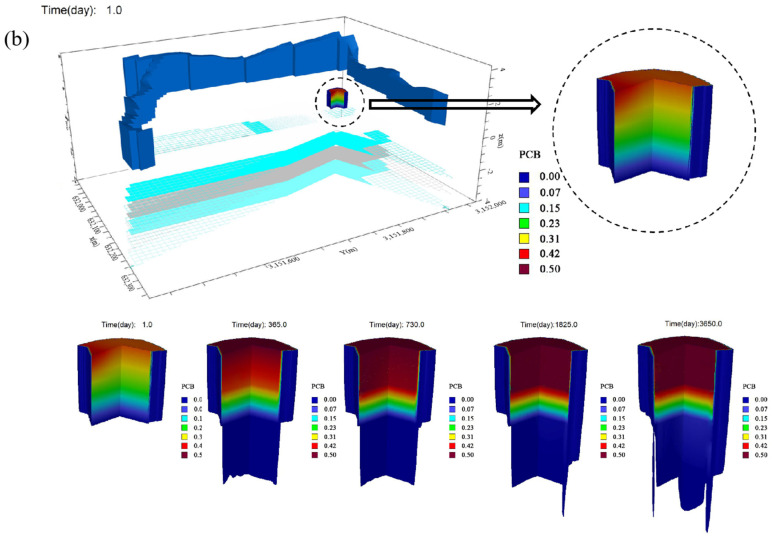
Three-dimensional map of Pb-PCBs composite pollutant transport based on numerical simulation: (**a**) map of pollution transport when Pb was the primary pollutant (the initial Pb concentration was 10 mg L^−1^); (**b**) map of pollution transport when PCB was the main pollutant (the initial PCBs concentration was 0.50 mg L^−1^) in the complex pollutants. The time point of each map was day 1, day 365, day 730, day 1825, and day 3650.

Understanding the migration process and trend of heavy metal and organic pollutants at the soil-water interface helped people develop targeted ways to prevent compound pollutants spread. The most convenient and economical way was to carry out numerical simulation on the VM software according to the results of the pollution characteristics of the contaminated site investigation, and seized the key indicators of the barrier measures to design the specifications of the cutoff wall in the upcoming field test. Considering Pb and PCBs had the risk of widespread pollution distribution in soil–groundwater environment, high super-standard concentration, and migration and diffusion in different media, we set Pb or PCBs as the initial pollutants in the VM software to make up for the different scenarios in which the numerical simulation could not fully reflect the heavy metal-organic combined pollution of the site and which pollutant concentration was relatively high at different environmental interfaces (Figure 5). Results of VM software numerical simulation (Figure 5) showed that no matter Pb or PCBs was the primary pollutant at the initial time, the longitudinal migration did not change significantly from year 5 to year 10 with the total migration depth reaching 1 m (Figure 5). The spread of pollution was fastest in the first year with the contaminated area reaching from 838 m^2^ to 1648 m^2^, while the expanded contaminated area during the second year was only one-fifth that of the first. By the end of 10th year, the concentration of pollutants in the pollution central area reached 9.70 mg L^−1^ with the total polluted area reached 2260 m^2^.

Generally, the migration speed of the compound pollutants was not fast, which could be ascribed to the low permeability (vertical hydraulic conductivity was 1.19 × 10^−7^ cm/s, horizontal hydraulic conductivity was 9.61 × 10^−8^ cm/s) of the polluted site (Table 1). In a simulation system of 10-year migration sport, the speed of both longitudinal and lateral diffusion was fast in the first year but much slower during the following years.

### 3.3. Effectiveness Validation of Cutoff Wall Based on Numerical Simulation

The service life of cutoff wall was investigated by setting the concentrations of Pb and PCBs in Grade IV standard value of China’s national groundwater quality standard as the breakthrough concentrations of pollutants on the cutoff wall. Breakthrough time referred to a criterion for judging the durability of the cutoff wall. Results indicated that the protocol of wall 3 (50 m × 12 m × 3 m) was best with its service time longest in the case of uniform breakdown concentration, no matter Pb or PCBs as the main pollutant in the model (Table 2; Figure S6). In this system, different sizes of cutoff walls all played important roles in pollutant barrier, with the order of action was: wall 3 > wall 4 > wall 2 > wall 1 (Figure S6). In general, cutoff wall design scheme with the size of 50 m × 2 m × 3 m was considered to have significant effects against the transport of Pb-PCBs compound pollutants. The above analysis also indicated that the critical factor in this pollution control system was the thickness of cutoff wall, rather than the length, that determined the stability and persistence of the barrier system.

### 3.4. Site Application of Cutoff Wall and Environmental Implications

The selected cutoff wall construction process included guide wall excavation, sodium bentonite waterproof blanket (total mass per unit area ≥ 4800 g/m^2^) laying, cutoff wall back-filling. The backfill material was modified clay, which was mixed by 5% modified bentonite and local pollution-free deep soil. Among them, the modified bentonite was Fe-modified sodium bentonite, and the Fe^3+^/Fe^2+^ concentration was 7.5 mol/kg.

**Figure 6:**
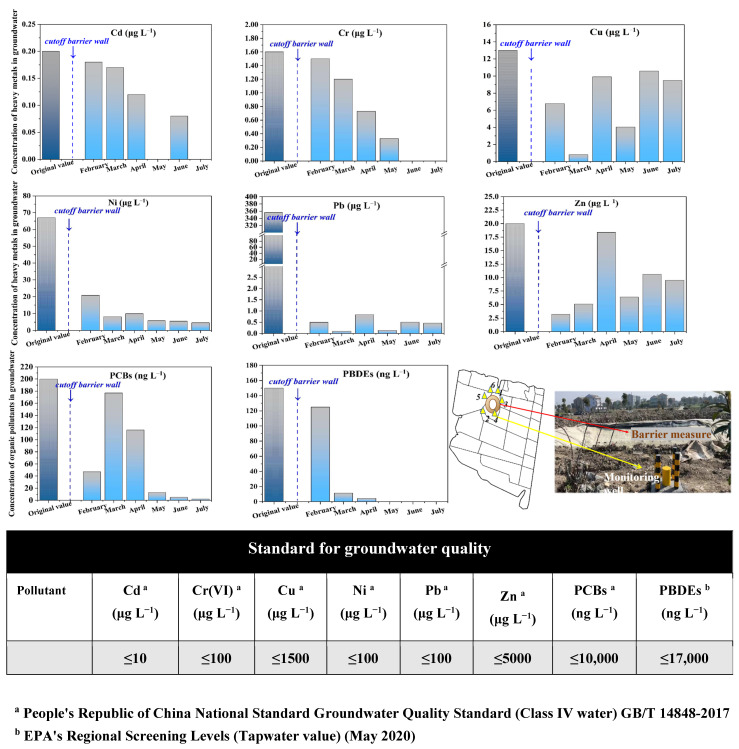
Concentration variations of main pollutants in the groundwater of site 2 over six-month after implement of cutoff walls. Pollutants included heavy metals (Cd, Cr, Cu, Ni, Pb and Zn) and organic pollutants (PCBs and PBDEs).

After applying the 50 m × 2 m × 3 m cutoff wall in site 2 according to the above technical steps, the environmental effect of cutoff wall engineering measures were reevaluated by groundwater monitoring for more than six months. During the six-month period, we monitored the spread of pollution by setting up monitoring wells around cutoff walls (Figure S5) and sampling groundwater continuously each month for chemical test (Figure 6). Results indicated that the original exceeded Pb concentration rapidly decreased under the action of the cutoff wall (down to 0.50 mg L^−1^), which lasted over six-month and always stayed at 1.00 mg/L (Figure 6). Other heavy metals did not exceed the groundwater quality standard during the six-month test, and basically continuous declined. Among them, the concentration of Cd and Cr(VI) in the groundwater declined to below detection limit. The average concentration of Ni continuously decreased from February to July and finally remained at 4.60 µg L^−1^. Cu and Zn decreased from original value (before applying cutoff walls) to 9.48 µg L^−1^ and 0.50 µg L^−1^, which were all below Grade IV groundwater standard value (Figure 6, [34] GB/T 14848-2017). The concentration of PCBs was also reduced compared to original value since the cutoff wall was implemented. However, it experienced a rising and then falling from March to April, which was probably ascribed to climate warming from winter to spring during the March. The final mean concentration remained at 2.10 ng L^−1^ in July. Comparatively, PBDEs concentration declined continuously from February to July after cutoff wall application and never exceeded the standard value (Figure 6).

The field-scale test confirmed the reliability of the numerical simulation results—the scheme of wall 3 (50 m × 2 m × 3 m) was appropriate for blocking the spread of compound pollution, which also verified the site adaptability and durability of cutoff walls. Since groundwater quality monitoring wells were set outside all around the cutoff wall, while the contaminated site was wrapped around the inside of the cutoff wall (Figure S5), the groundwater samplings from monitoring wells were considered as representatives of the environmental samples after cutoff wall working. Environmental indicators (including heavy metals and organic pollutants), did not exceed Grade IV standard value of China’s national groundwater quality standard, which indicated that cutoff wall engineering measures not only successfully blocked the compound pollutants in the contaminated site, also did not cause adverse effects on the surrounding environment.

## 4. Conclusions

The phenomenon of heavy metal-organic compound pollution (Pb, Cd and PCBs, PBDEs) was typical in the e-waste dismantling legacy site in Taizhou city, Zhejiang Province of China, in both soil and groundwater to different extent, but more severe at the topsoil. Xinhe village, with greater polluted area and diffusion potential, was taken an example for further studying the diffusion rule of compound pollutants at the soil-water interface. By predicting the transport path of Pb and PCBs in a 10-year period through numerical simulation, wall 3 (50 m × 2 m × 3 m) was selected for site application since its service time was predicted to be longest. Consecutive six-month groundwater monitoring showed that heavy metal and organic pollutants did not exceed national groundwater quality level IV standards after the implementation of the above cutoff wall technique. Through the field test, it was confirmed that the cutoff wall technology and its engineering measures were important means to prevent the spread of composite pollution, which also provided a new strategy for the environmental protection of contaminated sites.

## Figures and Tables

**Table 1:** Basic information on soil hydrogeological condition.

	Soil Sampling Points	Sampling Depth (m)	pH	Electrical Conductivity (μs/cm)	Soil Particle Composition					Soil Organic Matter (g/kg)	Dissolved Organic Matter (mg/kg)	*K_v_* Vertical Hydraulic Conductivity (cm/s)	*K_h_* Horizontal Hydraulic Conductivity (cm/s)
					Silt (%)	Sand (%)	Clay (%)	Gravel (%)	Break Stone (%)				
Site1	27 (topsoil)	0–−0.15	7.73	132	13.1	80.4	6.50	/	/	56.3	51.5	4.20 × 10^−8^	4.48 × 10^−8^
	18 (deep soil)	−1.5–−3.0	7.12	39.2	26.9	7.10	0	35.2	30.8	2.83	/		
Site2	11 (topsoil)	0–−0.15	7.70	179	42.1	7.60	50.3	/	/	40.8	41.89	1.19 × 10^−7^	9.61 × 10^−8^
	3 (deep soil)	−1.5–−3.0	7.96	60.8	/	/	/	/	/	2.97	/		
Site3	9 (topsoil)	0–−0.15	7.67	262	8.40	86.6	5.00	/	/	61.2	53.49	3.21 × 10^−8^	3.85 × 10^−8^
	3 (deep soil)	−1.6–−3.8	/	/	/	/	/	/	/	49.0	/		

**Table 2:** Service time and maximum breaking concentration of cutoff walls.

Cutoff Wall (Length × Width × Height)		Pb		PCBs	
		Breakthrough Time (Year)	Maximum Concentration (mg L^−1^)	Breakthrough Time (Year)	Maximum Concentration (mg L^−1^)
wall 1	50 × 1 × 3 m	10.91	1.020	14.96	0.051
wall 2	50 × 1.5 × 3 m	11.32	0.946	15.63	0.047
wall 3	50 × 2 × 3 m	14.14	0.883	19.33	0.044
wall 4	55 × 1.5 × 3 m	12.15	0.946	16.65	0.047

## Data Availability

Data are contained within the article and Supplementary Information.

## Supplementary Materials

The following supporting information can be downloaded at: https://www.mdpi.com/article/10.3390/toxics13010011/s1, Figure S1: Geological section map of sampling points in site 2; Figure S2: Groundwater flow map of contaminated site in different seasons; Figure S3: Overview of simulation area and grid division diagram of site 2 in the mathematical model; Figure S4: The fitting condition of groundwater flow model; Figure S5: Monitoring wells around the barrier wall were set up for groundwater quality monitoring and groundwater sample testing; Figure S6: The service time of cutoff walls.

## References

[1] Lin S.Y., Chen X.W., Cai Z.W., Shi J.B., Fu J.J., Jiang G.B., Wong M.H. (2022). Remediation of emerging contaminated sites due to uncontrolled e-waste recycling. Chem. Eng. J..

[2] Widmer R., Oswald-Krapf H., Sinha-Khetriwal D., Schnellmann M., Böni H. (2005). Global perspectives on e-waste. Environ. Impact Assess. Rev..

[3] Bakhiyi B., Gravel S., Ceballos D., Flynn M.A., Zayed J. (2018). Has the question of e-waste opened a Pandora’s box? An overview of unpredictable issues and challenges. Environ. Int..

[4] Shen C.F., Huang S.B., Wang Z.J., Qiao M., Tang X.J., Yu C.N., Shi D.Z., Zhu Y.F., Shi J.Y., Chen X.C. (2008). Identification of ah receptor agonists in soil of e-waste recycling sites from taizhou area in china. Environ. Sci. Technol..

[5] Shen C.F., Chen Y.X., Huang S.B., Wang Z.J., Yu C.N., Qiao M., Xu Y.P., Setty K., Zhang J.Y., Zhu Y.F. (2009). Dioxin-like compounds in agricultural soils near e-waste recycling sites from Taizhou area, China: Chemical and bioanalytical characterization. Environ. Int..

[6] Tang X.J., Shen C.F., Shi D.Z., Cheema S.A., Khan M.I., Zhang C.K., Chen Y.X. (2010). Heavy metal and persistent organic compound contamination in soil from Wenling: An emerging e-waste recycling city in Taizhou area, China. J. Hazard. Mater..

[7] Xing G.H., Wu S.C., Wong M.H. (2010). Dietary exposure to PCBs based on food consumption survey and food basket analysis at Taizhou, China—The World’s major site for recycling transformers. Chemosphere.

[8] Zheng J., Chen K., Yan X., Chen S., Hu G., Peng X., Yuan J., Mai B., Yang Z. (2013). Heavy metals in food, house dust, and water from an e-waste recycling area in South China and the potential risk to human health. Ecotoxicol. Environ. Saf..

[9] Awasthi A.K., Wang M.M., Awasthi M.K., Wang Z.S., Li J.H. (2018). Environmental pollution and human body burden from improper recycling of e-waste in China: A short-review. Environ. Pollut..

[10] Wang Y., Tian C., Wang Z., He D., Wu N., Zhang H., He S., Pan L., Ying C. (2021). Health risk and temporal trend of dietary potentially toxic elements exposure in the residents of the Shenzhen metropolis, China, between 2005 and 2017: A risk assessment based on probabilistic estimation. Environ. Geochem. Health.

[11] Lin S., Ali M.U., Zheng C., Cai Z., Wong M.H. (2022). Toxic chemicals from uncontrolled e-waste recycling: Exposure, body burden, health impact. J. Hazard. Mater..

[12] Jia K.Y., Qiao W.F., Chai Y.B., Feng T., Wang Y.H., Ge D.Z. (2020). Spatial distribution characteristics of rural settlements under diversified rural production functions: A case of Taizhou, China. Habitat Int..

[13] Gu Z.P., Feng J.L., Han W.L., Wu M.H., Fu J.M., Sheng G.Y. (2010). Characteristics of organic matter in PM2.5 from an e-waste dismantling area in Taizhou, China. Chemosphere.

[14] Liu J., Shi L.F., Du Y.P., Luo X.T., Hu P.J., Wu L.H., Luo Y.M., Christie P. (2024). Water-dispersible colloids facilitate the release of potentially toxic elements from contaminated soil under simulated long-term acid rain. Sci. Total Environ..

[15] Xia J., Wang J., Zhang L., Wang X., Yuan W., Peng T., Zheng L., Tian W., Feng X. (2022). Migration and transformation of soil mercury in a karst region of southwest China: Implications for groundwater contamination. Water Res..

[16] Xue S.G., Ke W.S., Zeng J.Q., Tabelin C.B., Xie Y., Tang L., Xiang C., Jiang J. (2023). Pollution prediction for heavy metals in soil-groundwater systems at smelting sites. Chem. Eng. J..

[17] Xiang Z.J., Wu S.J., Zhu L.Z., Yang K., Lin D.H. (2024). Pollution characteristics and source apportionment of heavy metal(loid)s in soil and groundwater of a retired industrial park. J. Environ. Sci..

[18] Leung A.O.W., Luksemburg W.J., Wong A.S., Wong M.H. (2007). Spatial distribution of polybrominated diphenyl ethers and polychlorinated dibenzo-p-dioxins and dibenzofurans in soil and combusted residue at Guiyu, an electronic waste recycling site in southeast China. Environ. Sci. Technol..

[19] Li N., Chen X.W., Deng W.J., Giesy J.P., Zheng H.L. (2018). PBDEs and Dechlorane Plus in the environment of Guiyu, Southeast China: A historical location for E-waste recycling (2004, 2014). Chemosphere.

[20] Xu C., Zhang Q., Gao L.R., Zheng M.H., Qiao L., Cui L.L., Wang R.H., Cheng J. (2019). Spatial distributions and transport implications of short- and medium-chain chlorinated paraffins in soils and sediments from an e-waste dismantling area in China. Sci. Total Environ..

[21] Zhai Y.Z., Jiang Y., Cao X.Y., Leng S.Y., Wang J.S. (2022). Valuation of ecosystem damage induced by soil-groundwater pollution in an arid climate area: Framework, method and case study. Environ. Res..

[22] Yang S., Gu S., He M., Tang X., Ma L.Q., Xu J., Liu X. (2020). Policy adjustment impacts Cd, Cu, Ni, Pb and Zn contamination in soils around e-waste area: Concentrations, sources and health risks. Sci. Total Environ..

[23] Wu Y., Li X., Yu L., Wang T., Wang J., Liu T. (2022). Review of soil heavy metal pollution in China: Spatial distribution, primary sources, and remediation alternatives. Resour. Conserv. Recycl..

[24] US EPA (2012). Institutional Controls: A Guide to Planning, Implementing, Maintaining, and Enforcing Institutional Controls at Contaminated Sites.

[25] US EPA (2013). Superfund Remedy Report.

[26] UK EA (2009). Dealing with Contaminated Land in England and Wales: A Review of Progress from 2000–2007 with Part 2A of the Environmental Protection Act.

[27] UK EA (2016). Dealing with Contaminated Land in England: Progress from April 2000 to December 2013 with Part 2A of the Environmental Protection Act 1990.

[28] Ministry of Ecology and Environment of the People’s Republic of China (2017). National Soil Pollution Situation Detailed Inspection of Soil Sample Analysis Test Method Technical Regulations. https://www.mee.gov.cn/gkml/hbb/bgth/201711/W020171106339408983483.pdf.

[29] (2018). Soil Environmental Quality Risk Control Standard for Soil Contamination of Development Land (Trial Implementation).

[30] (2015). Soil and Sediment—Determination of Polychlorinated Biphenyls (PCBs)—Gas Chromatography Mass Spectrometry.

[31] US EPA (2018). Method 8270E Semivolatile Organic Compounds by Gas Chromatography/Mass Spectrometry.

[32] Li R., Ren W., Teng Y., Sun Y., Xu Y., Zhao L., Wang X., Christie P., Luo Y. (2021). The inhibitory mechanism of natural soil colloids on the biodegradation of polychlorinated biphenyls by a degrading bacterium. J. Hazard. Mater..

[33] Xiang L.L., Song Y., Bian Y.R., Sheng H.J., Liu G.X., Jiang X., Li G.H., Wang F. (2016). A purification method for 10 polybrominated diphenyl ethers in soil using accelerated solvent extraction-solid phase extraction. Chin. J. Anal. Chem..

[34] (2017). Standard for Groundwater Quality.

