# Supplementary material for: Design, Implementation and Environmental Impact of Cutoff Wall for Pollution Control in an Industrial Legacy Site
Source: Toxics. 2024 Dec 25;13(1):11. doi: 10.3390/toxics13010011 (PMC11769112; doi:10.3390/toxics13010011)
Supplement: Supplementary file 1 [file toxics-13-00011-s001.zip › toxics-3326621-supplementary.pdf]

Supporting information

Detailed procedures of organic pollutant concentration test

a. Pretreatment and extraction of PCBs, PBDEs

The soil samples were freeze-dried and then sieved through a 0.25-mm mesh. For PBDEs extraction and determination, standards (^{13}C-BDE-209) was spiked. After about two grams of soil sample was homogenized with 4.0 g of diatomite and transferred to the extraction cell covered with silica sand, followed by accelerated solvent extraction (ASE) with hexane: acetone (4:1, v/v) at 100 °C and 1500 psi.

b. Purification of PCBs, PBDEs

PCBs, PBDEs extract of ASE was subsequently concentrated to 1 mL in a rotary evaporator at 42 °C.

For PCBs purification, the residue from rotary evaporation was purified with an extraction (SPE) column filled with 0.7 g of silica gel, 0.6 g of neutral alumina, 0.6 g of acid alumina and 0.6 g of anhydrous Na_2SO_4 from bottom to top. Before the purification of PCBs, the extraction (SPE) column was activated twice with 3 mL of n-hexane, then the column was further eluted with 5 mL of n-hexane. The collected eluate was dried under a stream of N_2 and finally re-dissolved in 1 mL of n-hexane.

For PBDEs purification, the residue from rotary evaporation was purified with an extraction (SPE) column filled with 0.5 g of anhydrous Na_2SO_4, 1.0 g of acid silica gel, and 1.0 g of anhydrous Na_2SO_4 from bottom to top. Before the purification of PBDEs, the extraction (SPE) column was activated twice with 3 mL of n-hexane, then the column was further eluted with 5 mL of n-hexane. The collected eluate was dried under a stream of N_2 and finally re-dissolved in 1 mL of hexane, then 50μL of PCB209 was added (final concentration of 10 ppb).

c. Instrumental analysis of PCBs, PBDEs

PCBs: GC7890 gas chromatograph (Agilent Technologies, Santa Clara, CA) was used to analyze PCBs concentrations. The chromatographic column was an HP-5 (30.0 m × 0.32 mm × 0.25 μm) with high purity nitrogen (50 mL min^{-1}) as the carrier gas and an injection volume of 1.0 μL. The injector and detector temperatures were 250 °C and 300 °C, respectively.

PBDEs: GC7890 gas chromatograph (Agilent Technologies, Santa Clara, CA) was used to analyze PBDEs concentrations. The chromatographic column was an HP-5 (30.0 m × 0.32 mm × 0.25 μm) with high purity nitrogen (50 mL min-1) as the carrier gas and an injection volume of 1.0 μL. The injector and detector temperatures were 250 °C and 310 °C, respectively. For BDE 209, the chromatographic column was an HP-5 (15 m × 250 μm × 0.1 μm), the injector and detector temperatures were 265 °C and 300 °C, respectively.

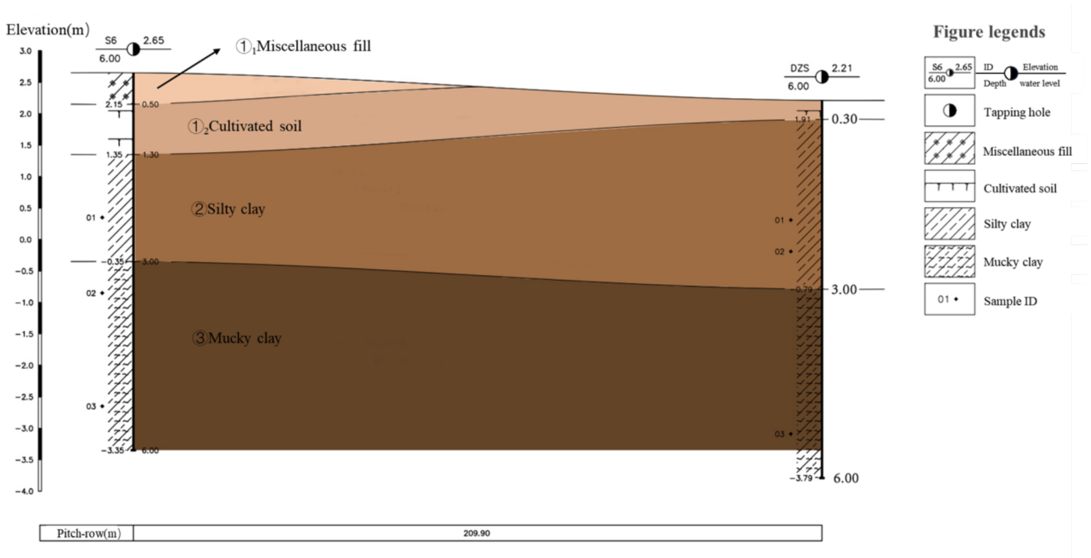

Figure S1. Geological section map of sampling points in *site 2*.

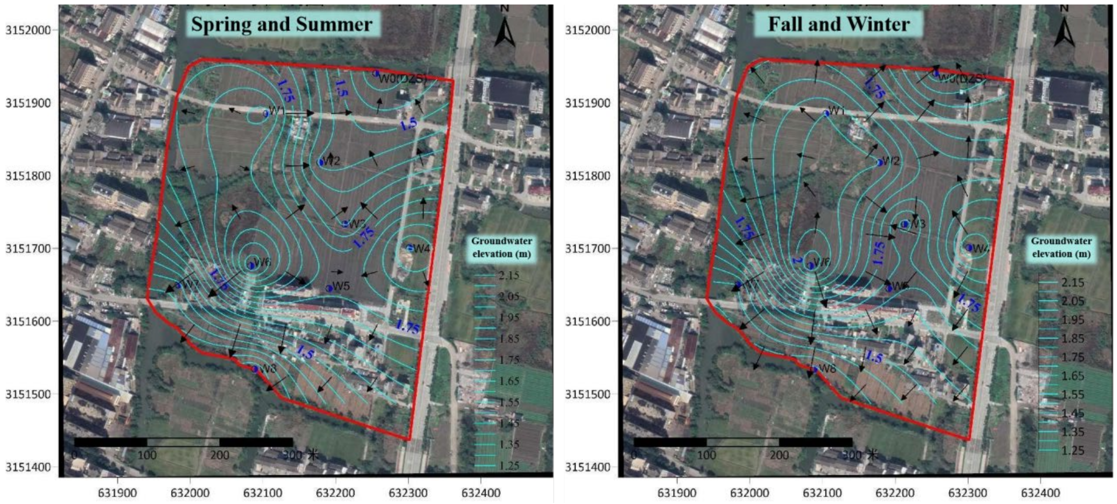

Figure S2. Groundwater flow map of contaminated site in different seasons.

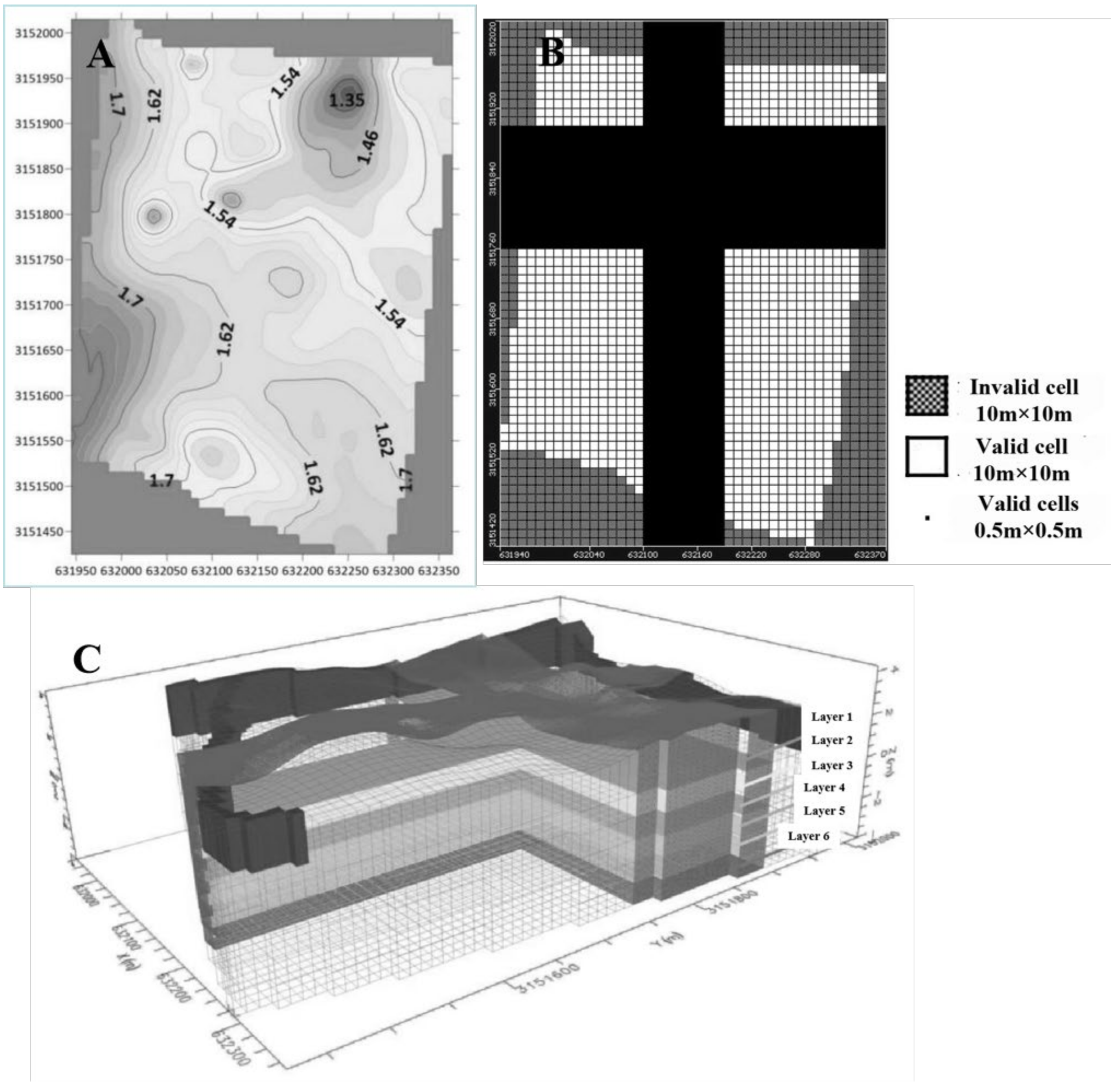

Figure S3. Overview of simulation area and grid division diagram of *site 2* in the mathematical model. A. Initial flow field in the simulated area; B. Grid division of simulation area; C. Stratigraphic grid division.

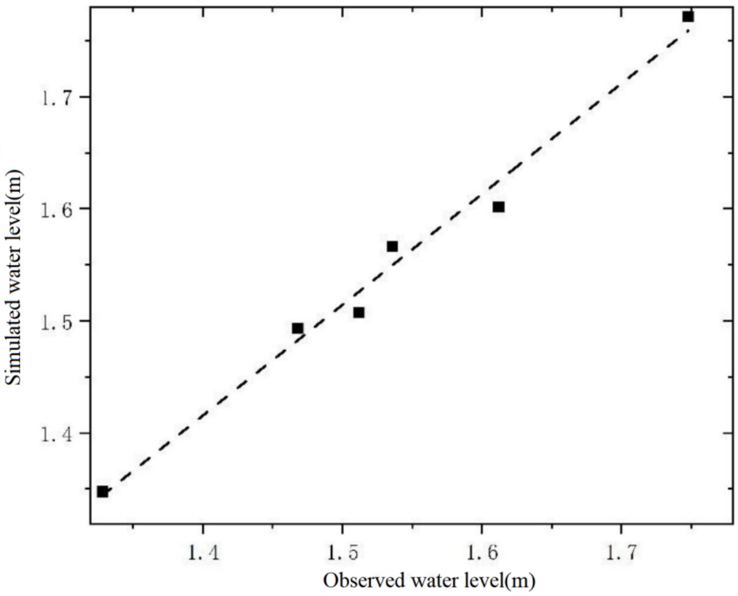

Figure S4. The fitting condition of groundwater flow model

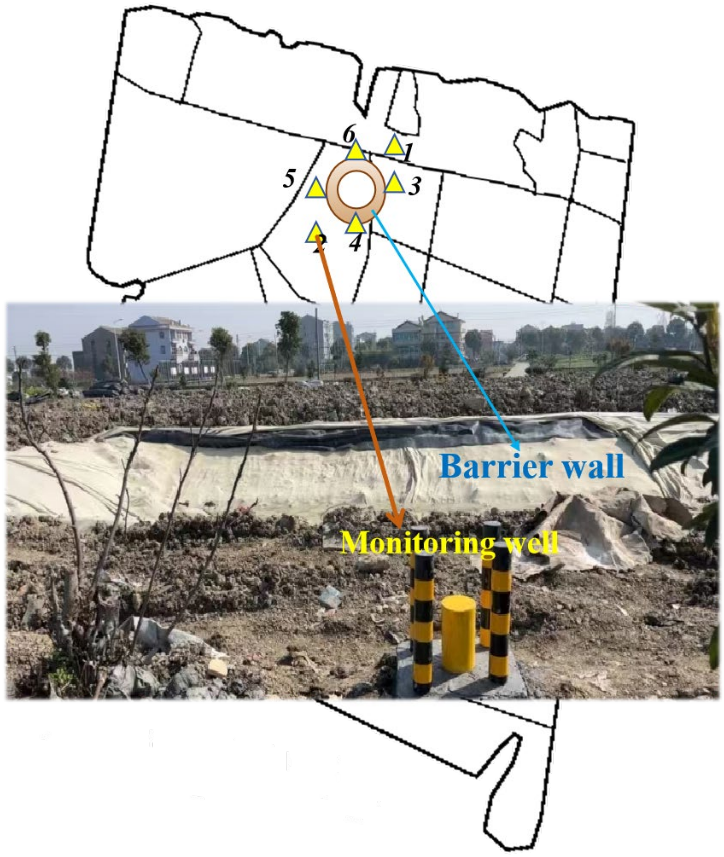

Figure S5. Monitoring wells around the barrier wall were set up for groundwater quality monitoring and groundwater sample testing. A total of six monitoring wells around the four directions outside of cutoff wall.

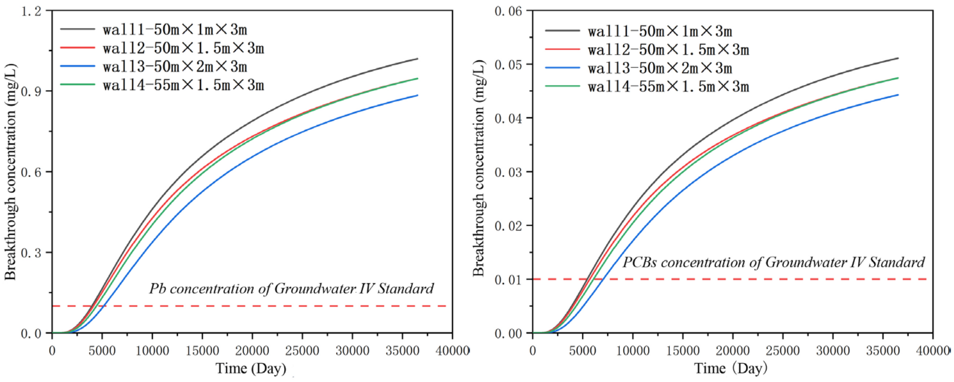

Figure S6. The service time of cutoff walls.
